# Unruptured Intracranial Aneurysm Treatment Score Concordance for Unruptured Aneurysm Treatment at a Quaternary Center

**DOI:** 10.7759/cureus.114719

**Published:** 2026-08-18

**Authors:** Isadora S Fonseca, Larissa N Costa, Claudionor N Segundo, Selma S Sabra, Ricardo A Gepp, Romel Corecha Santos

**Affiliations:** 1 Neurological Surgery, Escola Superior de Ciências da Saúde, Brasília, BRA; 2 Neurological Surgery, Hospital Sarah Kubitschek, Brasília, BRA; 3 Neurological Surgery, University of Colorado School of Medicine, Aurora, USA; 4 Neurological Surgery, University of Alabama at Birmingham, Birmingham, USA

**Keywords:** aneurysm size and treatment outcome, aneurysm treatment, comparison, intracranial aneurysm management, risk factors for aneurysms, treatment decision-making, uiats validation

## Abstract

Objective

This retrospective study analyzed records from a quaternary hospital’s multidisciplinary team (2002-2020) to investigate patients diagnosed with incidental intracranial aneurysms and their treatment approaches.

Methods

Clinical decisions (surgery vs. conservative) were compared with retrospectively calculated Unruptured Intracranial Aneurysm Treatment Score (UIATS) recommendations to identify variables influencing treatment divergence.

Results

Agreement between clinical decisions and UIATS was 69.3% (n=70), though Cohen’s kappa (0.25) indicated only fair statistical agreement. Discordance was significantly associated with aneurysm diameter (p=0.002), size (p=0.007), morphology (p<0.0001), multiplicity (p<0.0001), and risk scores (p=0.008). Notably, 10.9% (n=11) of patients received conservative care despite UIATS recommending surgery, while 19.8% (n=20) underwent surgery despite conservative recommendations.

Conclusion

Clinical decisions showed fair agreement with the UIATS. Discrepancies were primarily driven by specific aneurysm characteristics and evolving treatment protocols over the 18-year study period, highlighting that while UIATS is a valuable support tool, individualized multidisciplinary evaluation remains essential.

## Introduction

Aneurysms are abnormal dilations in blood vessels, generally in regions unable to withstand hemodynamic pressure, and may be ruptured or unruptured [1,2]. Eighteen percent (18%) occur in the anterior cerebral artery and its branches, 35% in the middle cerebral artery, 42% in the internal carotid artery (including the posterior communicating artery, responsible for 10% in isolation), and 5% in the vertebrobasilar arteries [3,4].

The prevalence of unruptured intracranial aneurysms (UIA) is 3.2% (mean age 50 years), higher in women and generally following a benign course. UIA ≤7 mm are usually asymptomatic, being diagnosed mainly by CT angiography and MR angiography. Without treatment, rupture carries high lethality (12% immediate death, 30% within one month, 25-50% within six months), and 30% of survivors become dependent [5-8].

Management of unruptured aneurysms includes conservative management, endovascular treatment, or surgical clipping, with the choice depending on aneurysm-related factors, patient factors, and associated patient anxiety [4,5,9]. Conservative management is indicated for lesions <7 mm in patients ≥60 years, except in cases with a strong family history of subarachnoid hemorrhage or a symptomatic aneurysm, with monitoring to detect growth and elective management as needed. Not all cases are suitable for invasive treatment (e.g., atherosclerosis, location), which sustains controversy over the best approach. Endovascular treatment has fewer complications but a slightly higher risk of post-procedural rupture [10-12].

Two primary studies described the natural history of unruptured cerebral aneurysms: the International Study of Unruptured Intracranial Aneurysms (ISUIA) showed increased rupture risk with diameter and that, for lesions <10 mm, surgical risks outweigh rupture risk, with limitations due to short follow-up; the Unruptured Cerebral Aneurysm Study (UCAS) associated morphology with rupture risk [12,13].

Given this decisional complexity, the Unruptured Intracranial Aneurysm Treatment Score (UIATS) was proposed to standardize management, developed through rounds of Delphi consensus, incorporating expert perspectives from a large international cohort. The UIATS generates two scores - one favoring definitive treatment and one favoring conservative management - whose difference should guide the decision, without replacing individualized clinical judgment, in order to bring management strategy closer to consensus for patients with unruptured intracranial aneurysms [14-16].

In practice, the therapeutic decision for UIA is frequently made by a multidisciplinary team, integrating neurosurgeons, neurologists, and interventional radiologists, whose judgment may diverge from the formal UIATS result. Evaluating this concordance is relevant for validating the scale as a decision-support tool and for identifying to what extent factors not captured by the model, such as specific characteristics of particular populations, for example patients in rehabilitation, influence the actual management adopted. This study retrospectively examines the application of UIATS in this context, comparing it with multidisciplinary decision-making and analyzing characteristic variables of these centers, such as stroke history, which may impact morbidity and therapeutic decision-making.

## Materials and methods

This research was approved by the institution’s ethics committee. The study was performed in accordance with the 1964 Helsinki Declaration and its later amendments. Because the data were anonymized and the study was retrospective, informed consent was waived.

**Figure 1:**
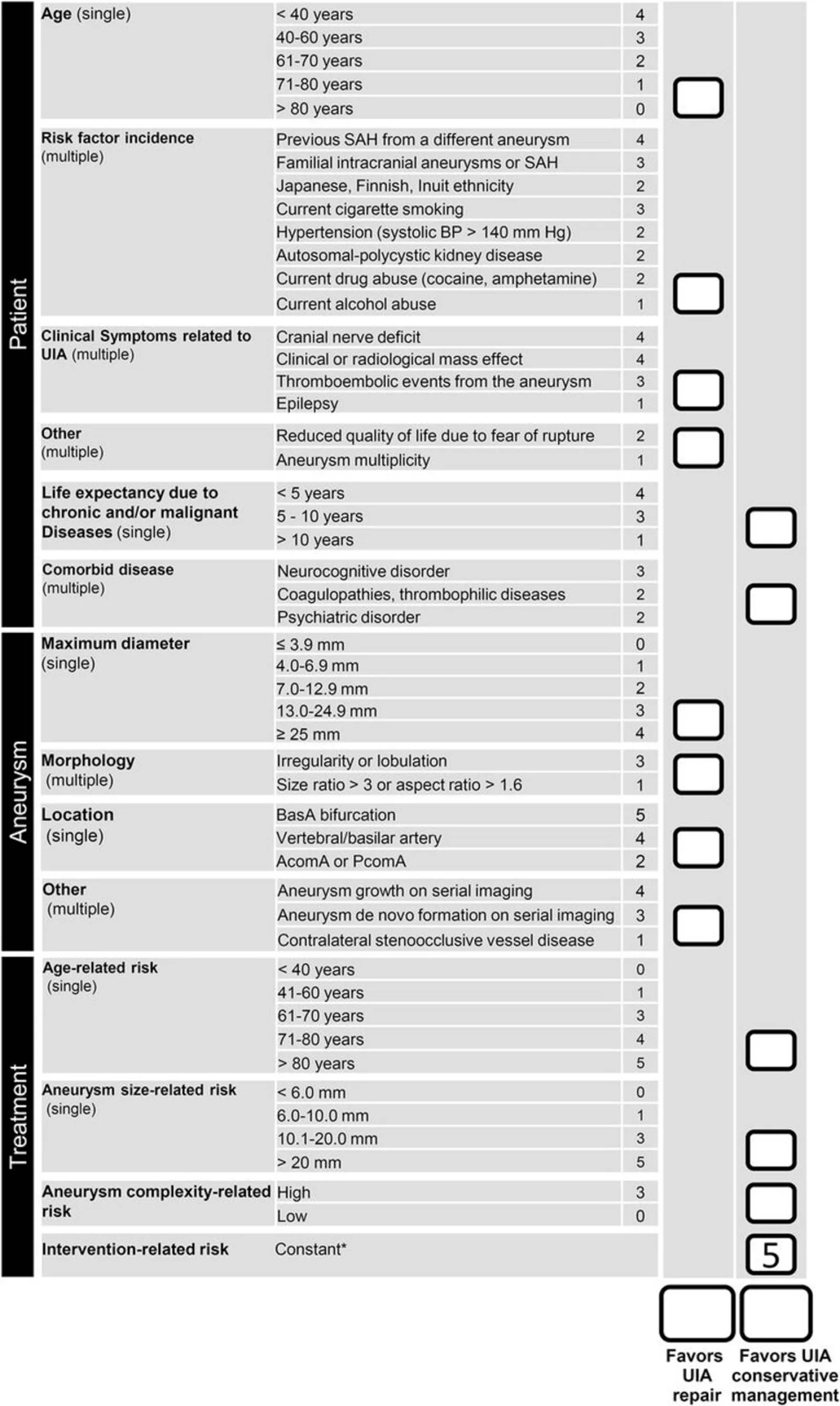
UIATS Source: Reproduced from Etminan et al. (2015) [6]. Available under CC BY-NC-ND 4.0. UIATS: Unruptured Intracranial Aneurysm Treatment Score

This is a retrospective cohort study comparing agreement with the UIATS scale (Figure 1). By reviewing medical records, patients admitted to the studied hospital between 2002 and 2020 with incidental unruptured intracranial aneurysms were selected. Inclusion criteria comprised patients aged 18 years or older, admitted to the quaternary center between 2002 and 2020, with a confirmed diagnosis of UIA established by neuroimaging, and who possessed all necessary medical record data for retrospective application of the UIATS. Due to the institutional characteristics of our center, the detected aneurysms were incidental findings; this was specifically defined as an unruptured aneurysm discovered fortuitously during diagnostic workup in a patient admitted or hospitalized for an unrelated clinical reason or pathology. Exclusion criteria applied to pediatric patients and individuals with missing data that precluded the proper completion of the scoring questionnaire. Patients with symptomatic aneurysms were also excluded, as well as aneurysms of non-saccular etiology (including mycotic, traumatic, fusiform, dissecting, or arteriovenous malformation-associated lesions) and patients with a history of prior surgical or endovascular treatment for the evaluated aneurysm.

The data were anonymized and entered into spreadsheets. Data on the UIATS scale, as well as the medical team's treatment choice for each case, were obtained from medical records and imaging examinations. This information was entered into SPSS for Windows (V17.0, 2008, SPSS Statistics for Windows, Version 17.0. Chicago: SPSS Inc.), from which the UIATS-suggested treatment was calculated. Data processing was performed by an institutional statistician to guarantee the protection and confidentiality of patient data.

Cases were separated into surgical and conservative approaches, subsequently evaluated using UIATS, and further subdivided into cases of agreement and disagreement between the team’s management and UIATS. This classification yielded five groups: (1) agreement between surgical treatment by the team and UIATS - “surgically treated according to the score”; (2) surgical treatment by the team but conservative recommendation by UIATS - “surgically treated in disagreement with the score”; (3) agreement on conservative management by the team and UIATS - “conservatively treated according to the score”; (4) conservative management by the team and surgical indication by UIATS - “conservatively treated in disagreement with the score”; and (5) cases indeterminate by UIATS.

For continuous variables (age, number of aneurysms, and follow-up), the normality of the distribution was assessed using the Shapiro-Wilk and/or Kolmogorov-Smirnov tests. Variables exhibiting a normal distribution (e.g., age) were compared using the independent-samples t-test. In contrast, those with a non-normal distribution (e.g., number of aneurysms and follow-up months) were compared using the nonparametric Mann-Whitney U test.

Regarding the categorical variables (e.g., Agerelatedrisk, Morphology), the Pearson's chi-squared test was utilized. In cases where the assumptions of the chi-squared test were not met, Fisher's exact test was applied instead. This test determines whether a significant association exists between two categorical variables and is ideally suited to small samples, as in this study.

Analysis via Fisher's exact test yielded p-values indicating which specific UIATS variables were most significantly associated with discrepancies between the medical team's decisions at the quaternary institution and the treatment proposed by the scale. P-values below 0.05 were considered indicative of statistical significance.

Additionally, the kappa coefficient was calculated to assess inter-rater agreement. In the context of this study, this represented the concordance among the neurosurgeons' decisions as documented in the retrospectively reviewed medical records.

## Results

In total, 101 patients were investigated, with 68 (67.3%) undergoing conservative aneurysm treatment and 33 (32.7%) undergoing surgical treatment. The average patient age in the study was 58.13 years. The follow-up period for these patients was 38.86 months (33.71 months for females and 56.30 months for males). The agreement between these evaluators must be taken into consideration. The proportion of agreement (p0) stood at 70 (69.3%).

Although the raw percentage agreement between historical clinical decisions and the UIATS recommendations was 69.3%, Cohen’s kappa coefficient was 0.25 (Table 1). According to the established Landis and Koch criteria, a kappa value within the 0.21-0.40 range represents only a 'fair agreement'. This notable discrepancy between a relatively high raw agreement and a low kappa value is a well-documented statistical phenomenon that occurs when there is a high probability of agreement occurring purely by chance due to the distribution of management choices. Clinically, this gap is highly relevant: it demonstrates that while nearly 70% of treatment decisions coincided, the structured, systemic concordance between real-world practice over 18 years and the UIATS algorithm remains modest, highlighting the heavy reliance on individual neurosurgical judgment and evolving care paradigms rather than standardized scoring guidelines.

**Table 1:** Decision concordance index Legend: “-” indicates an empty cell.

Unruptured Intracranial Aneurysm Treatment Score	Conservative	Repair	Total	Kappa	P-value
Conservative	57 (56.4%)	20 (19.8%)	77 (76.2%)	0.25	0.01
Repair	11 (10.9%)	13 (12.9%)	24 (23.8%)	-	-
Total	68 (67.3%)	33 (32.7%)	101 (100%)	-	-

There was a discrepancy between UIATS recommendations and the treatment approach adopted by the medical team. In the initial analysis, we assessed whether patients were receiving treatment by comparing their scores with those of others. Findings revealed that 11 (10.9%) of patients with a surgical recommendation by UIATS were receiving conservative treatment; they were considered conservatively treated, in disagreement with the score. Conversely, 20 (19.8%) of patients initially recommended conservative treatment by UIATS were surgically treated, contradicting the score. We sought to understand which factors were affecting these particular decisions. Based on the Fisher index, the following criteria were deemed significant for the discrepancy in UIATS outcomes: maximum aneurysm diameter (p=0.002), size (p=0.007), morphology (p=<0.0001), number of aneurysms (p=<0.0001), and risk assessment (p=0.008) (Table 2). Additionally, the presence of very low patient counts (zeros or ones) in certain risk factor subcategories resulted in data sparsity. Although Fisher’s exact test was appropriately utilized to accommodate small sample sizes, the reliability of the resulting p-values in these specific sparse subgroups should be interpreted with caution, which represents a statistical limitation regarding our data density (Table 2). 

**Table 2:** Baseline characteristics of patients, grouped by conservative and repair treatment Legend: “-” indicates an empty cell

	Conservative group	Repair group	P value
Total patients	68	33	-
Age (years)	58.1 (55.4 -60.8 )	51.6 (47.6 - 55.6)	p=0.01
Age (years)	-	-	p=0.059
< 40	4 (5.9%)	5 (15.2%)	-
41-60	30 (44.1%)	19 (57.6%)	-
61-70	27 (39.7%)	9 (27.3%)	-
71-80	7 (10.3%)	0 (0%)	-
Life expectancy (years)	-	-	p=0.092
< 5	1 (1.5%)	0 (0%)	-
5-10	7 (10.3%)	0 (0%)	-
>10	58 (85.3%)	33 (100%)	-
Aneurysm size (mm)	-	-	p=0.007
< 6 mm	53 (77.9%)	15 (45.5%)	-
6-10 mm	11 (16.2%)	13 (39.4%)	-
10.1-20 mm	2 (2.9%)	3 (9.1%)	-
> 20 mm	2 (2.9%)	2 (6.1%)	-
Maximum diameter (mm)	-	-	p=0.002
< 3.9	26 (38.2%)	3 (9.1%)	-
4.0-6.9	32 (47.1%)	16 (48.5%)	-
7.0-12.9	6 (8.8%)	10 (30.3%)	-
13-24.9	2 (2.9%)	3 (9.1%)	-
> 25 mm	2 (2.9%)	1 (3.0%)	-
Morphology	-	-	p <0.0001
0	38 (55.9%)	5 (15.2%)	-
Aspect ratio > 1.6	26 (38.2%)	21 (63.6%)	-
Lobulation (3)	3 (4.4%)	2 (6.1%)	-
Lobulation and aspect ratio > 1.6	1 (1.5%)	5 (15.2%)	-
Number of aneurysms	1.309 (1.1 - 1.5)	1.394 (1.1 - 1.6)	p <0.0001
Aneurysm complexity	-	-	p=0.266
High	20 (29.4%)	7 (21.2%)	-
Low	48 (70.6%)	26 (78.8%)	-
Risk factor	-	-	p=0.008
0	28 (41.2%)	10 (30.3%)	-
1	0 (0%)	1 (3.0%)	-
2	22 (32.4%)	9 (27.3%)	-
3	8 (11.8%)	2 (6.1%)	-
4	4 (5.9%)	0 (0%)	-
5	3 (4.4%)	8 (24.2%)	-
6	1 (1.5%)	2 (6.1%)	-
7	1 (1.5%)	0 (0%)	-
8	0 (0%)	1 (3.0%)	-
9	1 (1.5%)	0 (0%)	-

Regarding aneurysm size, aneurysms smaller than 6 millimeters and between 6 and 10 millimeters exhibited the most significant discrepancy between UIATS and the medical team approach, primarily leaning towards surgically treated, in disagreement with the score. The conservatively treated group, in disagreement with the score index, remained consistent across both categories. For aneurysms smaller than six millimeters, 5 (5%) were conservatively treated in disagreement with the score, and 8 (7.9%) were surgically treated in disagreement with the score. For those with sizes between six and ten millimeters, 5 (5%) were conservatively treated in disagreement with the score, and 9 (8.9%) were surgically treated in disagreement with the score. These results suggest that aneurysms up to 10 millimeters (Table 3) tend not to receive adequate treatment, leading to indications for surgical repair that are in disagreement with the score.

**Table 3:** Impact of aneurysm size on the decision index

Aneurysm size (mm)	Conservatively treated according to the score	Conservatively treated in disagreement with the score	Surgically treated in disagreement with the score	Surgically treated according to the score	Total
<6.0	48 (47.5%)	5 (5.0%)	8 (7.9%)	7 (6.9%)	68 (67.3%)
6.0-10.0	6 (5.9%)	5 (5.0%)	9 (8.9%)	4 (4.0%)	24 (23.8%)
10.1-20	2 (2.0%)	0 (0.0%)	1 (1.0%)	2 (2.0%)	5 (5.0%)
>20	1 (1.0%)	1 (1.0%)	2 (2.0%)	0 (0.0%)	4 (4.0%)
Total	57 (56.4%)	11 (10.9%)	20 (19.8%)	13 (12.9%)	101 (100.0%)

## Discussion

Previous scales, such as PHASES, developed in 2014 [10], have been used to evaluate unruptured aneurysms. PHASES is an acronym for six critical factors considered in this assessment: Population, Hypertension, Age, aneurysm Size, prior subarachnoid Hemorrhage, and aneurysm Location. It was designed to predict the five-year rupture risk of UIA by analyzing these specific factors [10]. This tool aimed to assess aneurysm rupture risk and assist in determining the need for surgical intervention. Criticism arose due to the exclusion of essential variables, such as smoking and family history of subarachnoid hemorrhage (SAH) [17,18]. In contrast, UIATS, which also assesses risk factors for UIA rupture, includes elements not considered in PHASES [18]. It quantitatively addresses patient-related, aneurysm-related, and treatment-related factors, offering a more comprehensive assessment of the variables influencing UIA treatment decisions. However, its appropriate use requires careful evaluation by professionals, with its findings serving as an aid in assessing the patient's condition rather than being used in isolation for predictive treatment planning [18].

Criticism of the UIATS scale focuses on its tendency to provide standardized opinions without concrete data to support decisions regarding the treatment of unruptured intracranial aneurysms (UIA) [18]. It is important to note that this study addresses, for the first time, the application of the UIATS scale in a tertiary institution focused on rehabilitation. This highlights the multiple ways in which the scale can be applied, analyzing variables that had not been previously addressed and that may impact morbidity, such as stroke history in therapeutic decision-making. This omission may have affected patients treated conservatively who disagreed with the score. With 23 patients having suffered a prior ischemic stroke and four having suffered a prior hemorrhagic stroke, there appears to be a deficiency in the scale's assessment for this specific group.

The research suggests that UIATS can be used as a therapeutic decision-support tool. The discrepancies, which are concentrated in maximum aneurysm diameter, size, morphology, number of aneurysms, and risk assessment, may be associated with aneurysm size. Aneurysms smaller than 10 millimeters appear to be more prone to overtreatment, implying that smaller aneurysms may present less clear and definitive results according to UIATS. On the other hand, aneurysms larger than 10 millimeters appear to be easier to assess using this scale.

It is crucial to note that terms such as "surgical treatment in disagreement with the score" and "conservative treatment in disagreement with the score" do not imply that the medical team acted incorrectly, but rather reflect how the scale is applied. Furthermore, it is vital to recognize that the scale may generate inconclusive results, requiring ongoing clinical and surgical evaluation. Additionally, it should be noted that subjective factors, such as life expectancy, anxiety, and associated comorbidities, are not included in the score and have a significant influence on medical treatment decisions; this is an important confounding factor in the study.

Regarding the limitations of this study, sample size (N=101) precluded multivariate analyses. As this is a retrospective cohort study with an 18-year temporal bias, there is an intrinsic limitation regarding data quality and availability, since the researcher had no control over the original data collection, and this data may be incomplete or inaccurate. In addition, there is a bias related to patients coming from a tertiary institution specializing in neurological rehabilitation.

## Conclusions

UIATS serves as a tool for assessing patients with intracranial aneurysms in various situations. When compared with decisions made by professionals at a tertiary institution, the observed differences stemmed mainly from factors such as maximum aneurysm diameter, size, age, and risk assessment. When analyzing the decisions made by professionals at this tertiary institution between 2002 and 2020, it becomes evident that they generally align with UIATS. This study offered a perspective on the application of the scale in a tertiary hospital, showing how a prior stroke influences medical decisions. Furthermore, it revealed that patients undergo more surgical treatments than the scale suggests, indicating that patient context and rehabilitation affect less conservative decision-making. Superior postoperative follow-up at tertiary institutions is another factor that differentiates the application of UIATS. This reinforces the importance of using the scale as a support tool in medical practice, together with careful, individualized patient assessment.

This study suggests that the application of UIATS can be replicated across various neurosurgical contexts, including tertiary centers, a distinguishing feature of this study. In this sense, it is suggested that larger centers, whether tertiary or not, validate the scale to demonstrate its replicability in other scenarios and contexts, aiming to improve the treatment of unruptured aneurysms.
